# How anxiety induces verbal hallucinations

**DOI:** 10.1016/j.concog.2015.11.009

**Published:** 2016-01

**Authors:** Matthew Ratcliffe, Sam Wilkinson

**Affiliations:** a Department of Philosophy, University of Vienna, Austria; b Department of Philosophy, University of Durham, UK

**Keywords:** Anxiety, Anticipation, Inner speech, Verbal hallucination

## Abstract

Verbal hallucinations are often associated with pronounced feelings of anxiety, and it has also been suggested that anxiety somehow triggers them. In this paper, we offer a phenomenological or ‘personal-level’ account of how it does so. We show how anxious anticipation of one’s own thought contents can generate an experience of their being ‘alien’. It does so by making an experience of thinking more like one of perceiving, resulting in an unfamiliar kind of intentional state. This accounts for a substantial subset of verbal hallucinations, which are experienced as falling within one’s psychological boundaries and lacking in auditory qualities.

## Highlights

• Anxiety both triggers verbal hallucinations (VHs) and shapes their content.
• A current model that accounts for this in terms of failed anticipation is critiqued.
• An important subset of VHs arise from a particular anxious style of anticipation.
• This anxious anticipation makes an experience of thinking more like one of perceiving.

## 1 Introduction

In this paper, we offer an account of the relationship between a substantial subset of verbal hallucinations (VHs) and feelings of anxiety.^1^ It is widely acknowledged that VHs are heterogeneous. Variables include volume, auditory quality, number of voices, degree of personification, emotional tone, thematic content, mode of address (second- or third-person), level of control over voices, and level of distress associated with them (Larøi, 2006, McCarthy-Jones et al., 2014, Nayani and David, 1996). We focus specifically on VHs that have repeated insults, threats and terms of abuse as their thematic contents. Many studies report that the majority of ‘voice hearers’ report such contents, and often only such contents.^2^ Although frequently associated with schizophrenia diagnoses, these experiences also arise in several other psychiatric conditions, including post-traumatic stress disorder, psychotic depression, bipolar disorder and borderline personality disorder, as well as in non-clinical subjects (Johns et al., 2014). First-person reports of abusive, insulting or threatening voices in these populations have much in common (Aleman & Larøi, 2008, p. 78), and we offer an account of VHs that is consistent with their diagnostic non-specificity. We propose that certain VHs arise due to pronounced and pervasive social anxiety, of a kind that is common to several psychiatric conditions.

We begin by presenting the view that anxiety is not merely a consequence of VHs: it both *triggers* them and *shapes* their content. Then we examine a model of how this happens, according to which VHs result from an anxiety-induced failure to anticipate thoughts. We argue that lack of anticipation is neither necessary nor sufficient for VHs. Instead, we introduce the notion of an ‘emotional style’ of anticipation and focus on one such style: anxious anticipation. Our central claim is that anxious anticipation of one’s own thought contents generates VHs by making an experience of thinking that *p* more like one of perceiving that *p*.^3^ We will show how this serves to clarify what Stephens and Graham (2000) call the “alien quality” of VHs, something that is not always attributable to their seeming to originate in the external environment or to their having sensory properties much like those of veridical perceptions. Our account captures those VHs that are experienced as internal in origin and as lacking in auditory properties. Others, we concede, require different explanations. We conclude by briefly examining how our personal-level account ties in with subpersonal theories.

Our case throughout is principally philosophical. We develop a phenomenological account of anxious anticipation, one that is both independently plausible and compatible with various empirical findings concerning VHs. However, we also draw upon first-person testimonies, in order to illustrate and provide further support for some of our claims. The main source of testimony is an Internet questionnaire study, which we conducted in collaboration with colleagues.^4^ Participants were invited to provide open-ended, free-text responses to questions that included “Please try to describe your voice(s) and/or voice-like experiences”, “How, if at all, are these experiences different from hearing the voice of someone who is present in the room?” and “What kinds of moods or emotions are associated with your voices?” All respondents quoted here had psychiatric diagnoses.^5^ Our intention is not to suggest that the phenomenology can simply be ‘read off’ first-person reports, thus comprising straightforward empirical evidence for our account. Rather, these are testimonies to be interpreted.^6^ The account that we offer is not only consistent with their content but aids in their interpretation, helping to make sense of otherwise puzzling experiences that people struggle to describe. It is corroborated by its ability to do so.^7^

## 2 Verbal hallucinations and anxiety

VHs, especially those with unpleasant contents, are generally accompanied by depression and anxiety. It is easy to see how such an experience might cause anxious distress. However, anxiety and depression frequently arise before the onset of VHs, and anxiety is particularly prevalent among ‘voice-hearers’ in both clinical and non-clinical populations (Allen et al., 2005, Kuipers et al., 2006, Paulik et al., 2006). It is important to distinguish two findings: (a) generalised anxiety is present before voices arise, and (b) there is heightened anxiety immediately before and during the VH experience. We focus on (b), but will also suggest a role for (a). According to Delespaul, de Vries, and van Os (2002, p. 97), anxiety is the “most prominent emotion during hallucinations and reports of anxiety intensity exceeded baseline levels before the first report of auditory hallucinations”. It has also been hypothesised that increased anxiety both triggers VHs and shapes their content, although the mechanism remains unclear (Freeman & Garety, 2003, p. 923). This view is consistent with first-person descriptions of the emotional states that immediately precede VHs:

“It’s worse when I’m stressed, anxious or scared.” (#3)

“When I am feeling anxious they grow stronger. When I am alone as the day goes on they get stronger.” (#6)

“Fear, unsafety, scare, not knowing” (#19)

“Loneliness, depression, anxiety, feeling unloved, deserted, uncared for” (#28)

What is described is not simply ‘anxiety’ but ‘social anxiety’. Hence the individual often withdraws from others and may complain of feeling socially isolated and estranged (Hoffman, 2007).^8^ Romme, Escher, Dillon, Corstens, and Morris (2009) provide fifty detailed first-person accounts by voice hearers, which include numerous references to social vulnerability, anxiety, fear, social isolation, shame and feeling lost. Many add that their feelings of anxiety, depression and estrangement originated in distressing social relationships and traumatic events, including neglect and abuse during childhood. All of this is consistent with the prevalence of abusive and insulting voices; the content of VHs is usually mood-congruent (Larøi, 2006, p. 165).

That many VHs arise in a context of social anxiety and are immediately preceded by heightened anxiety does not, in itself, imply a causal relationship. However, the fact that treating the anxiety often leads to a reduction in VH frequency and severity lends further support to the hypothesis that anxiety is causally implicated in VHs (Kuipers et al., 2006, p. 28). Even so, *how* anxiety might cause VHs remains thoroughly unclear. In what follows, we will offer a personal-level account of how this happens.

## 3 Anxiety and anticipation

We think that the notion of ‘anticipation’ is central to understanding how anxiety induces VHs. However, contrary to the received view, we will argue that VHs are not attributable to a *lack* of anticipation but to *how* one anticipates. A recent phenomenological account that attributes VHs to anxiety and to a consequent lack of anticipation is offered by Gallagher (2005), who seeks to accommodate both VHs and thought insertion. (He does not say whether or how the two differ, an issue we will return to later.) We take this account as a starting point from which to develop our own position. Gallagher’s approach is premised on the view that acts of thinking incorporate experiences of anticipation. It is not that one anticipates thinking something before one thinks it. That would fall foul of an infinite regress objection: anticipating the thought that *p* is itself a thought with the content ‘the thought that *p*’, which would be anticipated by a further thought with the content ‘the thought that the thought that *p*’, and so on.^9^ What one anticipates is less determinate in content. Gallagher offers the analogy of listening to a melody, where one might not anticipate hearing a particular note before one hears it, but one has at least some sense of what will come next, as illustrated by the surprise one feels when a note is out of tune.^10^ By analogy, one might anticipate the thematic “gist” of a thought, the content of which is more determinate (Hoffman, 1986).

According to Gallagher, if a thought were not anticipated at all, it would arrive fully formed, rather than crystallizing out of something that is congruent with it but less determinate in content. This would amount to a sense of its coming from elsewhere, like the unanticipated and fully-formed communications we receive from other people. He further proposes that such experiences could occur due to “unruly emotions such as anxiety”. In brief, anxiety disrupts anticipation and, were it to immediately precede a particular thought, that thought would “appear as if from nowhere”; it would be “sudden and unexpected” (Gallagher, 2005, pp. 194–200). This is consistent with a widespread emphasis in the VH literature on prediction failure (e.g. Frith, 1992). Of course, the breakdown of a subpersonal mechanism that predicts (or, more specifically, ‘monitors’) the generation of thoughts is distinct from a personal-level prediction failure. However, the personal-level correlate of sub-personal prediction failure is often taken to be an experience of one’s thoughts as unanticipated. As Fletcher and Frith (2009, p. 56) put it, “an inner voice is unpredictable and therefore feels alien”.^11^

However, Gallagher’s account faces a serious problem, as does any other account that appeals to a lack of conscious anticipation. First of all, it is arguable that lack of conscious anticipation is not *sufficient* for VHs. Many thoughts appear to arise unanticipated, such as a song that suddenly starts ‘playing in one’s head’ or a seemingly random thought that does not cohere with the gist of one’s thinking and may also disrupt one’s train of thought. In response, perhaps even these thoughts are anticipated to at least some degree and therefore differ from VHs. It is difficult to arbitrate between conflicting phenomenological claims here. But, whatever the case, it can be added that lack of anticipation is clearly not *necessary* for VHs. In short, many voice-hearers *do* anticipate their voices, to the extent that they may be able to solicit a voice, dialogue with it and predict the thematic content of what it will ‘say’ next. In fact, it has been claimed that a majority of voice-hearers are able to converse with their voices (e.g. Garrett & Silva, 2003, p. 449). In one influential study, 51% reported that they had at least some control over their voices, 38% that they could initiate a voice, and 21% that they could stop a voice (Nayani & David, 1996, p. 183). Some also describe a feeling of ‘personal presence’ preceding a voice.

Hence we suggest that the simple contrast between anticipating and failing to anticipate is an unhelpful one; VHs do not arise due to lack of anticipation. Indeed, having a thought that is completely unanticipated might well be a fairly mundane experience, one that does not involve the relevant sense of externality. Instead, VHs arise when thoughts are anticipated in a distinctive *way*. All instances where one anticipates that *p* can be qualified in terms of the following:

1. Determinacy of content: *p* can be more or less specific.
2. Mode of anticipation: *p* can be anticipated as certain, uncertain, probable, improbable, doubtful, and so forth.
3. Emotional style of anticipation: the prospect of *p* can be the object of a range of different emotions, such as excitement, curiosity, hope or fear.

By appealing to a combination of [1] and [3], we will argue that VHs arise due to a distinctive emotional style, that of anxious anticipation. We will assume that the mode of anticipation, [2], is usually that of certainty or high probability.

## 4 Anxiety and the alien quality

We accept, as a premise, that many emotions either are intentional states or at least incorporate intentional states: *R* is afraid of *p*; *S* is guilty about *q*. This is not to imply that the intentionality of emotion is a matter of cognitive ‘judgment’ or ‘appraisal’ rather than ‘affect’ or ‘feeling’. There are various ways of arguing that some or all emotional feelings are themselves intentional, and that their objects are not restricted to one’s own bodily states (see, for example, Goldie, 2000, Prinz, 2004, Ratcliffe, 2008). We further maintain that types of emotion are not just *commonly* but *properly* associated with only certain other types of intentional state. For instance, feeling guilty about something is properly associated with remembering it but not with imagining it or anticipating its occurrence. And fearing something is properly associated with anticipating its occurrence but not with remembering that it has already occurred. One might feel guilty about something that has not happened, in a situation where one has already set the wheels in motion such that it almost certainly *will* happen. Even so, the guilt remains past-directed: one feels guilty about *p* in virtue of the fact that one remembers doing *q*, where *q* is likely to cause *p*. It is not psychologically impossible to fear what has already happened or to feel guilty about a merely imagined state of affairs. Nevertheless, the experience would be a strange one. In the case of guilt, one might then think ‘I am wrong to feel guilty about this’ or, alternatively, ‘maybe I am not just imagining doing it; maybe I actually did do it’.

When remembering that *p* is associated with feeling guilty about *p*, it is debatable how the two relate. Perhaps there is a singular kind of intentional state, that of ‘guiltily remembering that *p*’. Alternatively, a distinction might be drawn between two distinct and simultaneous intentional states with the content *p*. Or it could be that one first remembers *p* and then feels guilty about *p*; so guilt borrows its content from a preceding intentional state. The same applies to fearing and perceiving that *q*. However, even if the two experiences are – according to some criterion – distinct, it is plausible to maintain that they *affect* each other. Were one to feel persistent, intense, recalcitrant guilt about something merely imagined, one’s imagining having done *p* might take on some of the qualities of memory; *p* would start to *feel* like something one had actually done. More generally, we propose the following: where ‘emotion *x* with content *p*’ is properly associated with ‘intentional state *y* with content *p*’ but not with ‘intentional state *z* with content *p*’, its association with *z* can result in *z*’s taking on some of the phenomenological characteristics of *y*. In extreme cases, the result is a novel kind of experience, one that is of neither *y* nor *z*. This, we will argue, is how anxiety induces VHs. It is not properly associated with one’s own thought contents and, when it is associated with them, they are experienced as the contents of a perception-like intentional state that also retains some of the features of thought.

Our proposal requires further clarification. It is commonplace to think something and also feel anxious about it. Hence one might object that thoughts clearly are proper objects of anxiety. However, what we are ordinarily anxious about is *p*, not having the thought that *p*. For example, where *p* is ‘I might lose my job’, I am anxious about actually losing my job, not about having the thought that I might. Feeling anxious about ‘the thought that *p*’ is a more unusual experience. But how could this account for VHs? There is a sense in which anxiety is intrinsically ‘alienating’ or ‘externalising’. It presents its object – however determinate – as something unpleasant that one is confronted with. The object of anxiety is something that threatens, something that one feels helpless in the face of.

It is important to distinguish different senses of ‘externality’ here. To say that an object of anxiety is essentially external is not to insist that it be experienced as physically external to one’s bodily boundaries. Our own bodily experiences can be objects of anxiety. A person who fears she has a serious medical condition may become increasingly anxious about certain persistent bodily sensations. And chronic illness can involve more widespread feelings of alienation from one’s body. It is encountered in a way that is strange and previously unfamiliar, as an actual or potential impediment to one’s activities and an object of anxiety, rather than something in which one has implicit ‘trust’ (Carel, 2013, van den Berg, 1966). Where an object of anxiety is physically external to oneself, one feels ‘alienated’ from it in a similar way. One could feel comfortably immersed in or uncomfortably separate from one’s physically external, interpersonal surroundings. When suffering from pronounced social anxiety, one does not simply feel physically separate from others. One experiences a different kind of separation from them; one is estranged from them, threatened by them, vulnerable and helpless. In this respect, anxiety is comparable to some experiences of pain. Consider an intense, lingering pain in one’s hand that persists independently of any external stimulus. The painful hand is not experienced as something ‘external’ to one’s body. But one feel alienated from it all the same, in the sense that one is confronted by something unpleasant, something one seeks to avoid but can do nothing about. Hence something can be experienced as external and alien or, alternatively, as internal and alien. The sense of alienation that we are concerned with here has nothing to do with perceived physical location.^12^ And neither does that which many voice-hearers describe, since the voices are often reported as internally located, and yet alien.

We suggest that certain VHs arise when one’s own thought contents become objects of anxiety and are thus experienced as ‘alien’. This is consistent with the observation that many voice-hearers dread their voices and, more specifically, what it is that the voices ‘say’. The distressing content is something the voice-hearer is confronted with, something she might try unsuccessfully to resist, to avoid: “it’s mocking me, I hate that one […] I am left in a state of fear […]. They don’t sound like me. They are angry most of the time. I don’t like to think of mean things, I try hard not to, but the more I try not to think the more the voices get nasty” (#22). Of course, it could be that the person experiences *p* and is subsequently anxious about it, due to the unpleasantness of *p* and also to *p*’s seeming to originate from elsewhere. We acknowledge this, but propose that causation goes both ways: anxious anticipation of content *p* can also generate an experience of *p* as alien. To illustrate how this happens, consider various familiar experiences that involve an indeterminate, affectively charged thought content coalescing into something more determinate. Take the realisation that you have left your bag on the train. As you depart from the station, this might begin as a surge of anxiety, the content of which can be roughly characterised as ‘something is wrong’; ‘I’ve not done something’ or ‘something important is missing’. This becomes ‘I’ve left something on the train’ and then ‘I’ve left my bag on the train’, after which the repercussions of what has happened becomes progressively clearer. Indeterminate content *p* arises, eliciting anxiety, and one anxiously anticipates the dawning of *q*, where *q* is a more determinate form of *p*. (The initial experience can also arise when nothing is wrong, in which case the content sometimes remains indeterminate, and the feeling fades upon recognition that all is well.)

The view that thought contents can increase in determinacy as they form is consistent with various proposed explanations of VHs. For instance, Fernyhough (2004) suggests that inner speech is more usually condensed and fragmented, and that the experience of externality is attributable to its anomalous re-expansion. And, according to the influential theory proposed by Hoffman (1986, p. 503), VHs are generated by disruption of a discourse planning process, which involves “abstract planning representations that are linked to goals and beliefs”. These give one a broad sense of what is coming next and precede more determinate contents. More generally, the emphasis that much of the VH literature places on ‘inner speech’ suggests a process of some kind whereby thoughts are converted into inner speech (Stephens & Graham, 2000, p. 81). The received view is that VHs involve experiencing one’s own ‘inner speech’ as non-self-produced, rather than one’s thoughts *per se*, where inner speech is construed as a medium in which only some of our thoughts appear. Hence thought content could provoke anxiety, the object of which is the subsequent content of inner speech. For current purposes, we do not need to endorse a specific theory of what happens or how it happens. All we need commit ourselves to is the claim that thought content *p* precedes thought content *q*, where *q* is a more determinate form of *p*.

Now, it could be argued that the content of the thought remains the same throughout such a process, that it is simply ‘translated’ into inner speech. However, whatever the process we are referring to might turn out to consist of, we suggest that it does at least involve differing degrees of content determinacy. That this is so becomes clearer once we emphasise the emotional content of VHs. Many emotions are intentional states with contents that can be conveyed in linguistic form. However, even if one were to insist that they incorporate some kind of ‘propositional content’ from the outset, this is not the same as their incorporating inner speech. It has been argued that spoken language does not just serve to convey pre-formed emotional states but also to individuate or even partly constitute them, at least in some instances (e.g. Campbell, 1997, Colombetti, 2009). Amongst other things, language can gives an emotion a more specific content. Similar points are made by the phenomenologist Maurice Merleau-Ponty (1962, pp. 177–182), for whom speech increases the determinacy of thought (and emotion): “the most familiar thing appears indeterminate as long as we have not recalled its name”; “the clearness of language stands out from an obscure background”.^13^ It is informative to revisit Hoffman (1986) in the light of these reflections. His approach has a cognitive emphasis throughout. The abstract plans and goals that enable discourse planning have – it appears – a propositional structure, although their content is less specific than that of inner speech. But consider an example Hoffman uses to illustrate his point. When asked to describe where she lives, a patient says the following:

Yes, I live in Connecticut. We live in a 50-year-old Tudor house. It’s a house that’s very much a home….ah…. I live there with my husband and son. It’s a home where people are drawn to feel comfortable, walk in, let’s see….a home that is furnished comfortably – not expensive – a home that shows very much my personality. (Hoffman, 1986, p. 506)

The overall theme that constitutes a sense of ‘where things are heading’ is a consistently emotional one.^14^ Utterances are not just ‘mood-congruent’; what we have is the *articulation of* an emotion or mood, something that renders its content increasingly specific. Now, let us assume that ‘inner speech’ can play a similar role to spoken language. Thus, in the case of an abusive ‘voice’, there is an unpleasant emotional content *p*, which provokes anxious anticipation of a more determinate linguistic content *q*, one that is elicited by *p* and consistent with *p*. Anxiety is intrinsically alienating and so its object, the thought that *q*, is experienced as alien, as something unpleasant that one faces and is unable to avoid. Whatever forms of anticipation our thinking more usually involves, anxious anticipation of thought content is not one of them. That style of anticipation is more typical of certain affectively charged perceptual experiences. So an unfamiliar, perception-like experience of thought content arises.

It might be objected that the thought content ‘I’ve left my bag on the train’ is not experienced as alien and that the process sketched here therefore fails to account for the ‘alien quality’ of VHs. But there is a crucial difference between the two. In the train case, one is anxious about the *fact* that one has left one’s bag on the train, not about the *thought* content ‘I’ve left my bag on the train’. However, in the case of a VH with the content ‘you’re a worthless piece of filth’, the thought content is itself an object of anxiety. The way it is anticipated as it coalesces thus renders it alien, something unpleasant before which one feels helpless. Hence an emphasis on lack of anticipation is misleading.^15^ To revisit the melody analogy, consider listening to a piece of music that involves a build-up of tension (an opera by Wagner, perhaps). One senses that something intrusive will blast in; it is on its way. And yet, when it arrives and conforms to the indeterminate expectation one had of it, it is still encountered as disruptive, as arising from elsewhere, set apart from the music that preceded it.

## 5 Interpreting first-person accounts

The approach we have outlined is consistent with empirical findings concerning (i) the thematic content of many VHs, (ii) the prevalence of anxiety, and (iii) the occurrence of similar kinds of VH experience in several psychiatric conditions and in non-clinical populations. In addition, it is consistent with many first-person testimonies and serves to further illuminate those testimonies. VH contents are sometimes explicitly described as linguistic manifestations of negative, self-directed emotional appraisals that are themselves sources of anxiety. For example:

“It’s hard to describe how I could ‘hear’ a voice that wasn’t auditory; but the words used and the emotions they contained (hatred and disgust) were completely clear, distinct and unmistakeable, maybe even more so that if I had heard them aurally. […]. I heard the voices of demons screaming at me, telling that I was damned, that God hated me, and that I was going to hell”.(#9)

This person further describes how the contents of his VHs “reflected all the judgmental attitudes I had heard from my family and church”. The emotional judgments are themselves feared; they are met with “anxiety/panic and incapacitating depression” (#9). Another questionnaire respondent remarks, “I hate everything about myself. […] I hear a voice that confirms everything I think about myself and sometimes it feels as if it is the only one that will tell me the real truth about myself”. She adds, “I can never concentrate on anything but how I am feeling and the voice I hear”. Thus, there is a combination of distressing, self-directed emotional appraisals and heightened attentiveness towards them; she waits for them to arrive.^16^ The content of her ‘voice’ is specifically associated with that of negative, self-directed emotions: “I took an anti-psychotic to stop the voice and it helped a bit as I don’t have something that seems so real confirming my feelings so outrightly”^17^. Reference to a voice ‘confirming’ one’s feelings indicates that it not only expresses pre-formed feelings but also adds to them in some way. This is plausibly construed in terms of its giving them a more determinate content, one that is an object of anxious anticipation. Feelings of inadequacy and the like are at first indeterminate, but can take on a more determinate linguistic guise that ‘confirms’ the emotional appraisal it is congruent with and out of which it arises.

Some first-person accounts further indicate a process of exactly the kind that we have described. An experience induces anxious anticipation, which then proceeds to shape the experience in question:

“Due to the murmuring voice experiences being so distressing with each successive occurrence however, I grew to dread ever more either whenever another experience would appear to possibly be forthcoming or, once in the midst of an actual ongoing experience, what would come next; waiting for the next shoe to drop.” (#31)

“It’s very difficult to describe the experience. Words seem to come into my mind from another source than through my own conscious effort. I find myself straining sometimes to make out the word or words, and my own anxiety about what I hear or many have heard makes it a fearful experience. I seem pulled into the experience and fear itself may shape some of the words I hear.” (#32)

“I have come to recognise the voices as expressions of anxiety, perhaps even a recognition of a fear I have about myself that I am not prepared to entertain as being part of my personality.” (#34)

There is anxious anticipation of what is coming next, rather than a lack of conscious anticipation (#31), which affects what is then experienced (#32). And, as indicated by (#34), anxiety about one’s own thought contents is also associated with a sense of their being alien; the anxiety is constitutive of one’s ‘disowning’ something distressing.

One might object that inner speech does not have auditory properties, regardless of whether or not its content is experienced as alien, whereas VHs are auditory experiences (e.g. Wu, 2012). Furthermore, VHs are often experienced as originating in the external environment. Nothing we have said explains their auditory properties or the fact that they are physically ‘external’, as well as ‘alien’ in our sense. We acknowledge that some VH experiences most likely conform to orthodox definitions of hallucination, according to which verbal hallucinations are auditory experiences that arise in the absence of appropriate external stimuli (e.g. Frith, 1992, Halligan and Marshall, 1996). Several accounts of VHs emphasise such an experience (e.g. Garrett and Silva, 2003, Leudar et al., 1997, Wu, 2012). However, others suggest that VHs are generally lacking in auditory qualities (e.g. Moritz and Larøi, 2008, Stephens and Graham, 2000). As Frith (1992, p. 73) suggests, a VH can involve something more abstract than hearing a voice, “an experience of receiving a communication without any sensory component”. The two views can be reconciled by acknowledging that VHs come in both guises. David (1994) states that most but not all subjects experience voices as arising “inside the head”, while Nayani and David (1996) report that 49% of their subjects heard voices through their ears, 38% internally and 12% in both ways. Leudar et al. (1997, p. 889) state that 71% of their subjects heard only internal voices, 18% heard voices “through their ears”, and 11% heard both. Internal VHs are not always described as bereft of auditory properties but, whatever auditory properties they might have, there is a substantial phenomenological difference between these two types. This is readily apparent when we turn to first-person descriptions by individuals who experience both:

“The voice inside my head sounds nothing like a real person talking to me, but rather like another person’s thoughts in my head. The other voices are to me indistinguishable from actual people talking in the same room as me.” (#1)

“There are two kinds – one indistinguishable from actual voices or noises (I hear them like physical noises, and only the point of origin (for voices) or checking with other people who are present (for sounds) lets me know when they aren’t actually real. The second is like hearing someone else’s voice in my head, generally saying something that doesn’t ‘sound’ like my own thoughts or interior monologue.” (#17)

Hence we suggest that it is fruitful to draw a broad, over-arching distinction between two subsets of VHs: (i) those that are experienced as external in origin and auditory in character; (ii) those that are experienced as internal in origin and lacking in auditory properties. Our account applies specifically to (ii). One might worry that what we have proposed conflicts with the observation that even internal VHs are usually described in terms of audition, rather than other kinds of perceptual experience. However, information of the relevant kind is usually received through auditory channels, at least in the absence of visual stimuli such as reading materials. So, even when it is bereft of the relevant sensory qualities, it lends itself to description in those terms. Furthermore, talk of hearing is often qualified. For example, references to the ‘sound’ of a voice and to ‘hearing’ might appear in scare quotes. Some of these internal ‘voices’ may not have any auditory qualities at all, a view that is consistent with reports of VHs in congenitally deaf subjects (e.g. Aleman & Larøi, 2008, pp. 48–9). Nevertheless, it is plausible to suggest that some of them do have audition-like properties. The claim that inner speech is sometimes or always *wholly* bereft of auditory properties is by no means uncontroversial. For example, Hoffman (1986) takes it to involve ‘auditory imagery’, and there may be considerable interpersonal variation too.

## 6 An unfamiliar kind of intentional state

Even though anxiety about one’s own thought contents is unusual, one might object that it is plausibly more widespread than the kind of VH experience we seek to account for. There could well be many people with disruptive, intrusive and self-directed thought contents that provoke anxiety but are not experienced as VHs. However, up to this point we have only emphasised one of two roles played by social anxiety, that of an immediate trigger for VHs. Generalised anxiety and social isolation are also disposing factors (which is not to rule out others). One experiences heightened anxiety about something specific in a context of already feeling more generally anxious and estranged. Depression and anxiety are both associated with what we might call ‘diminished agency’. One’s personal and interpersonal surroundings are globally oppressive and no longer invite effortless responses to meaningful possibilities in the way they once did. So there is a pervasive sense of being incapable of action, and even thoughts may seem sluggish, effortful, bereft of the kind of active anticipation that is involved when one is drawn into and absorbed in a theme (Benson et al., 2013, Ratcliffe, 2013). If the person feels more generally passive, helpless and incapable in the face of a threating world, the phenomenological distance between actively initiating something and receiving it from elsewhere may already be lessened. This could render her more vulnerable to a blurring of the phenomenological boundary between thinking that *p* and perceiving that *p*.

This is consistent with the observation that voices are generally “perceived as being extraordinarily powerful” (Birchwood et al., 2000, Chadwick and Birchwood, 1994). The individual’s relationship with her voices corresponds to her relationship with the social world; she feels passive and vulnerable in the face of interpersonal threat. There are also reports of inner dialogue becoming “more pronounced” before the onset of voices in some cases, with “subtle pre-psychotic distortions of the stream of consciousness – such as abnormal sonorization of inner dialogue and/or perceptualization of thought” (Raballo & Larøi, 2011, p. 163). This suggests a more general blurring of the experienced difference between kinds of intentional state, which would render one more prone to experiencing anxiety-inducing thought contents as alien. It also points to a gradual process, whereby the person becomes anxious about certain thematic contents, with raised anxiety leading to their progressive alienation, sometimes culminating in an experience of the voices as “almost personified” (Raballo & Larøi, 2011, p. 165).^18^

What we have outlined here also complements an approach to delusions proposed by Currie, 2000, Currie and Jureidini, 2001, according to which a delusion is not a recalcitrant false belief but an imagining that is mistaken for a belief. In the case of VHs, there is similarly confusion between two kinds of intentional state: perceiving and thinking. Currie and Jureidini (2001) construe this as an epistemic problem, where one actually imagines something but mistakes one’s imagining that *p* for the belief that *p*. However, they later reject a categorical distinction between imagination and belief, allowing for the possibility of intentional states that fall somewhere between the two (Currie & Jureidini, 2004).^19^ Whether or not our account of the blurring between thought and perception is an epistemic or constitutive one depends on which definitions of ‘perception’ and ‘thought’ one is working with.

Our emphasis is on the phenomenology of VHs. We have suggested that an *experience* of perceiving differs from one of thinking, and that anxious anticipation can lead to a perception-like experience of thought content.^20^ We grant that non-phenomenological conceptions of thought and perception could be adopted, which would re-cast the situation in epistemic terms. For example, if perception is *defined* as necessarily involving receipt of information from a sensory source, then a VH of the kind we have described is non-perceptual, pure and simple, and any first-person impression to the contrary is mistaken. However, in purely phenomenological terms, one does not mistake an experience of type *x* for one of type *y*; one has an intentional state that is neither *x* nor *y*: “it definitely sounds like it is from inside my head. It’s at some kind of border between thinking and hearing” (#18). The view that VHs involve an unfamiliar kind of experience, one that falls between thinking and perceiving, is supported by the observation that people frequently struggle to describe them. VHs are often described as ‘almost like’ something; it is ‘as though’ something were the case. For example, they might be described as ‘like’ telepathy:

“The commentary and the violent voices I heard as though someone was talking to me inside my brain, but not my own thoughts. Almost like how telepathy would sound if it were real. I don’t know how else to explain it.” (#4)

“..there are things I ‘hear’ that aren’t as much like truly hearing a voice or voices. [….] Instead, these are more like telepathy or hearing without hearing exactly, but knowing that content has been exchanged and feeling that happen.” (#7)

Given this, it is interesting to consider the possibility that certain kinds of VHs and what is called ‘thought insertion’ – which are traditionally treated as separate kinds of symptom – are in fact the same phenomenon (Ratcliffe and Wilkinson, 2015). It could be that there are varying degrees of ‘perceptualisation’, which lend themselves to description in terms of either VH or thought insertion. And it could also be that much the same experiences are described in different ways. A quasi-perceptual experience of thought content could be related in terms of (a) a perception with an anomalous content or (b) a thought content that is not one’s own.^21^

Acknowledging that VHs involve an unfamiliar kind of intentional state also casts light on the phenomenon of ‘double bookkeeping’. Many who voice delusional beliefs and describe hallucinatory experiences also speak and act in ways that distinguish their delusions from other beliefs and their hallucinations from veridical perceptions, thus suggesting different kinds of experience. Sass (1994, p. 3) describes this as follows:

Many schizophrenic patients seem to experience their delusions and hallucinations as having a special quality or feel that sets these apart from their ‘real’ beliefs and perceptions. […] Indeed, such patients often seem to have a surprising, and rather disconcerting, kind of insight into their own condition.

With specific reference to VHs, van den van den Berg (1982, p. 105) observes that voices are often given a “special name” to set them apart from perceptual experiences, due to their having a “recognizable character of their own which distinguishes them from *perception* and also from *imagination*”. This is complemented by our view that VHs are not quite like perceptions or thoughts, a view that also explains why the majority of clinical and non-clinical voice-hearers are readily able to distinguish their ‘voices’ from veridical auditory perceptions (Moritz & Larøi, 2008).

## 7 Varieties of VH

As we have emphasised, our account is intended to accommodate only some of those experiences that are labelled as ‘VHs’. Furthermore, it could be that ‘internal VHs that are bereft of at least some auditory properties’ are themselves heterogeneous. While we have emphasised anxious anticipation of inner speech, Michie, Badcock, Waters, and Maybery (2005) propose that VHs involve memory intrusions. Now, McCarthy-Jones et al. (2014) report that only 39% of their subjects acknowledged VH contents resembling memories and fewer still said that their VHs were memories. Even so, it could be that some internal VHs are like this. Indeed, internal VHs could encompass experiences of inner speech, memories and imaginings, as well as some contents that blend memories with imaginings. And the predominance of one form or another may reflect individual differences, different life histories and different diagnostic categories. For instance, we might find a predominance of alienated memory contents in cases where there is past trauma. However, inner speech VHs with less pronounced auditory qualities may be more common in schizophrenia, thus accounting for more frequent reports of ‘thought insertion’ in schizophrenia. However, this is not a problem for our account, given that what we have proposed need not be specific to the experienced boundaries between perception and inner speech. The alienating role of anxiety could be easily extended to the anticipation of distressing memories and imaginings, both of which may have more pronounced auditory qualities.

It is also worth keeping in mind that ‘social anxiety’ is not a singular phenomenon but something that would benefit from further analysis. Some first-person accounts emphasise shame and humiliation, others interpersonal threat and helplessness, and others guilt and self-hate. Different variants of social anxiety are likely to be associated with different thematic contents, given that VH contents are mood-congruent. Again, some of these differences may correspond to different psychiatric categories. For example, a person with a diagnosis of psychotic depression might hear voices that mock her and criticise her for her failures (Larøi, 2006). Hence the account we have offered has the potential to accommodate considerable phenomenological diversity, and to distinguish VH characteristics that are more typical of one or another diagnosis.

What about external VHs? If first-person accounts are to be taken at face value, they are sometimes much like veridical auditory perceptions. Hence they are unlike what we have described and also arise in a different way. However, social anxiety is implicated here too. Extreme social anxiety could dispose a person towards the anticipation of interpersonal communications with negative, self-directed contents. Delespaul, deVries and Van Os (2002) found that VHs are most likely to occur either when one is in the presence of lots of people or when one is alone. Building on this, Dodgson and Gordon (2009) propose, on the basis of clinical case-studies, a kind of VH called a ‘hypervigilance hallucination’ which occurs especially in ‘noisy’ environments where stimuli are susceptible to multiple interpretations. This, they suggest, accounts for a “substantial subset of externally located voices”.^22^ The existence of hypervigilance hallucinations as a separate subtype was subsequently supported by Garwood et al. (2013) who, based on a cluster analysis, showed that VHs tend to occur when (i) attention is directed *inward* in *quiet* contexts, and (ii) attention is directed *outward* in *noisy* contexts. This finding fits nicely with our account. In both internal (inner speech or memory-based) VHs and external, hypervigilance VHs, anxious anticipation shapes and distorts the experience. In instances of anxious hypervigilance, out of external stimuli (such as a ticking clock or the muffled sound of neighbours talking) will emerge the experience of a voice telling the subject exactly what the subject is afraid of hearing.

Thus anxiety could also be the underlying cause of at least some external VHs, even though internal and external VHs are generated in different ways. Our account also allows for in-between cases. For example, a perceptual stimulus might trigger an imagining, which is then experienced as an object of pronounced anxiety, and therefore as alien and perception-like. The same applies to inner speech: an external stimulus with auditory qualities could trigger an increasingly determinate linguistic content that is experienced as alien. Unlike an internal VH, this would seem to originate in the external environment, given its association with a perceived environmental cause. In such a case, auditory properties might also be ‘interpreted’ in such a way that they are consistent with the content of the communication.

Even so, our emphasis on anxiety cannot do justice to all VH experiences. Some VHs do not have distressing contents (Copolov, Mackinnon, & Trauer, 2004). Indeed, some voice-hearers obtain consolation, support and/or guidance from their voices. This applies to many of those VHs that arise in the context of grief. In a study of nearly 300 widows and widowers in Wales, Rees (1971) found that nearly half had hallucinations of the deceased spouse, sometimes lasting many years. Feelings of presence were most common, but VHs were also reported by 13%. Most of these people found their hallucinations comforting and helpful. We do not claim to have dealt with such cases, and we concede that the phenomenology of grief – in all its complexity – needs to be addressed separately. The same goes for various other kinds of VH experience. However, it may be possible to extend our general approach to VHs without the specific emphasis on anxiety. We have not claimed that anxious anticipation is the *only* way of anticipating one’s own thought contents that blurs the boundaries between intentional state types.

A further limitation of our account is that we have addressed only the ‘phenomenological’ or ‘personal’ level of description and have not postulated any associated mechanisms. What we have supplied here can, however, operate as an *explanandum* for neurobiological approaches. If one wants to provide a subpersonal account of how *x* is generated, where *x* is phenomenological in character, it helps to have a good account of what *x* consists of. That is what we have tried to provide, and there are clearly implications for accounts of the subpersonal mechanisms involved in VHs. According to a popular family of approaches, our nervous systems distinguish endogenous from externally produced stimuli through a process of self-monitoring. In particular, when a motor command is sent, a copy of that motor command is used to predict the sensory consequences of the action. When the actual sensory consequences match the predicted sensory consequences, the nervous system ‘judges’ that it is self-produced and sensory attenuation occurs (as if it were ‘saying’: ‘don’t worry about this: it’s only you’). When monitoring goes awry, sensory attenuation fails to occur and endogenous stimuli are erroneously attributed to an external cause (e.g. Campbell, 1999, Frith, 1992, Frith et al., 2000, Jones and Fernyhough, 2007, Seal et al., 2004). It is not clear how anxiety might fit into a self-monitoring story, as a cause rather than an effect of VHs. Furthermore, our anxiety-based account does not appeal to motor processes. Indeed, one might sense a tension between our account and the self-monitoring approaches, given that we emphasise a change in the style of anticipation, rather than a lack of anticipation. Of course, personal- and subpersonal-level explanations are to be distinguished from each other. And it could be that our anticipating *p* in style *x* rather than *y* involves the breakdown of certain subpersonal prediction mechanisms, while others continue to operate or perhaps operate differently. But, until a more specific account along such lines is developed, the claim that personal level anticipation in style *x* rather than *y* involves a breakdown of subpersonal prediction lacks explanatory power.^23^

## 8 Conclusion

We have argued that anxiety induces VHs in the following way: anxious anticipation of thought contents as they become increasingly determinate results in a quasi-perceptual experience of thought content. This is because anxiety is not properly associated with ‘the thought that *p*’; anxiety alienates us from its objects in a way that we are not ordinarily alienated from our own thought contents. Insofar as the person faces something that she seeks to avoid and is confronted by something that she feels helpless to resist, the resulting experience resembles an affectively charged perception more so than a mundane episode of thought. This account fits in well with subjective reports to the effect that anxiety triggers or aggravates VHs, that the voices confirm negative self-evaluations, and that the voices fall somewhere in between experiences of hearing and thinking. However, we also made clear that our account applies only to a subset of VHs, those with negative content, and most clearly to those that are experienced as internal in origin and lacking in auditory qualities.

## Footnotes

1 The more usual way of referring to the phenomena that we address here is ‘auditory verbal hallucinations’ (AVHs). We opt for the term ‘verbal hallucinations’ (VHs) instead since, as will become clear, we doubt that all VHs are auditory in character.

2 Nayani and David (1996, pp.182-6) state that, in their study, “the most commonly encountered hallucinated utterances occurring in 60% of the whole sample were simple terms of abuse” and that “vulgar expletives” were most frequent, while Leudar, Thomas, McNally, and Glinski (1997) report that 53% of their subjects heard abusive voices.

3 In maintaining that it is like something to *perceive* that *p*, which differs from what it is like to *think* that *p*, we are committed to the view that there is some kind of ‘cognitive phenomenology’. Hence one might object to our approach by drawing on arguments to the effect that there is no distinctively ‘cognitive’ experience. However, we take the line that the nature of cognitive phenomenology can be made clearer by engaging with phenomena such as VHs and that our account, insofar as it is plausible, constitutes a case for cognitive phenomenology. It would be a mistake to impose a conception of cognitive phenomenology (or its absence) on one’s subject matter from the outset. See the essays in Bayne and Montague (2011) for several different positions concerning the existence and nature of cognitive phenomenology.

4 The study was conducted as part of the Wellcome Trust funded project ’Hearing the Voice’ in 2013, by several members of the project. It received ethical approval from the Durham University Philosophy Department Research Committee. Study design was closely based on earlier work addressing the phenomenology of depression (for details, see Ratcliffe, 2015).

5 All responses quoted in this paper are numbered. Diagnoses are as follows: schizophrenia (#32, #34); schizoaffective disorder (#9); borderline personality disorder (#1, #3, #4, #6); psychosis (unspecified) (#7); bipolar disorder (#17, #19, #28); major depression (#6, #18, #31); PTSD (#22).

6 See Ratcliffe (2015, Chapter 1) for a detailed discussion of the methodological issues to be kept in mind when interpreting testimonies of this kind.

7 Our account is not consistent with every questionnaire response we received. However, we seek to accommodate a distinctive subset of VH experiences, rather than all such experiences. In the process, we also argue that other VH experiences are quite different. The questionnaire study was concerned with the full range of VH experiences.

8 Broome et al. (2005, p. 26) observe that psychosis is often preceded by depression, anxiety and social estrangement, and that they may have a causal role to play. However, we would add they are not specific to schizophrenia or to psychosis more generally, and neither are VHs.

9 This objection was first raised by Akins and Dennett (1986) against the account proposed by Hoffman (1986).

10 Gallagher also offers a more detailed account of temporal experience, which draws on the work of Edmund Husserl (e.g. Husserl, 1991).

11 Gallagher’s account has at least two advantages over that of Frith (1992). His claim that we anticipate thoughts does not require that there be a phenomenologically implausible “intention to think”, of the kind Frith appeals to. Gallagher also accounts for the fact that VHs tend to have consistent thematic contents: one anticipates the coming of a thought with more anxiety when its content is distressing.

12 The analogy is made more compelling by the observation that the somatic and affective components of pain are dissociable (Grahek, 2007, Radden, 2009). The sense of alienation we have described is attributable to the latter.

13 We also find something like this in the writings of some psychotherapists. For instance, Gendlin (2003, p. 38) claims that we have an inarticulate “felt sense” of a problem or situation. He remarks on the inchoate feeling that you have forgotten something or failed to do something and how, on some occasions, “suddenly, from this felt sense, it bursts to the surface”. He distinguishes the “felt sense” from emotions such as anxiety but his account is more generally consistent with the view that determinate linguistic contents can arise out of less determinate emotional contents.

14 In fact, in his response to commentaries, Hoffman (1986, p. 542) acknowledges that VHs can cause distress and increase “arousal”. He adds that “these increases in arousal could cause further disruptions in discourse planning and VHs. Thus, causality may be circular and difficult to tease apart”.

15 Although the account we have sketched here is a personal-level one, we also allow for the possibility that non-conscious anticipation of thought content could trigger anxiety and contribute to a subsequent experience of it as alien.

16 Our claim that VH contents are anxiously anticipated is thus consistent with the finding that VHs are associated with heightened “self-focus” (Allen et al., 2005).

17 This account is taken from a response to another questionnaire study, on experiences of depression. It was conducted in 2011 as part of the AHRC- and DFG-funded project ‘Emotional Experience in Depression’. This respondent (#16) had a diagnosis of severe depression with hyper-manic traits. For full details of the study, see Ratcliffe (2015).

18 A connection can also be made here with what Louis Sass calls ‘hyperreflexivity’, a heightened attentiveness to more usually unreflective aspects of experience, which is most fundamentally involuntary but can come to incorporate forms of voluntary attention as well. It results in a pervasive sense of alienation from one’s own psychological and bodily processes. According to Sass, it is in the context of this that more localised anomalous experiences, such as delusions and hallucinations, arise. Anxiety, of the kind of case we have described, amounts to a kind of hyperreflexivity. However, this is not to suggest that hyperreflexivity invariably takes such a form. For instance, there may be cases where hyperreflexive alienation from one’s own experiences and thoughts arises in some other way and *makes* one anxious. The anxiety would then feed into and exacerbate the original experience. There is also the question of whether certain kinds of experience are associated with certain diagnostic categories. Anxious alienation of thought content is consistent with several different diagnoses. However, we would not want to rule out the possibility that schizophrenia sometimes involves something more specific or qualitatively different from what we describe here. Sass’s own view is that, while the most disruptive forms of hyperreflexivity are associated with schizophrenia diagnoses, it also has a role in certain other conditions, including depersonalization disorder and dissociative conditions (Sass, 1992, Sass, 1994, Sass, 2014).

19 See also Gerrans (2014) for a recent approach to delusions that makes a non-phenomenological case for rejecting clear-cut distinctions between ‘belief’ and ‘imagination’.

20 Garrett and Silva (2003, p. 453) also suggest that VHs involve “a new category of experience that blends elements of perception and thought but remains distinct from both”. However, they emphasise the sensory qualities of VHs in a way that we do not.

21 See also Langland-Hassan (2008, p. 373) for the view that VHs are difficult to describe, given that they do not fit into familiar psychological categories, and that a VH may sometimes be an experience of thought insertion under another description.

22 This is also consistent with the “neural diathesis – stress” model of schizophrenia (Walker & Diforio, 1997), especially a more recent version of it that places the emphasis on responses to situations involving an “uncontrollable, social-evaluative threat” (Jones & Fernyhough, 2007, p. 1174).

23 In fact, Chris Frith, one of the principal architects of the self-monitoring approach, has more recently raised various concerns about it and begun to explore other explanatory models within a ‘predictive processing framework’ (PPF) (Fletcher and Frith, 2009, Frith, 2012). We think that the PPF may offer a more promising way of reconciling our anxiety-based account with a subpersonal-level explanation. According to the PPF, the brain ‘adopts hypotheses’ based on how well they predict inputs (or, to use the technical jargon, how well they minimise prediction error), and these hypotheses determine what the experience is like for the subject. This framework promises to be more in keeping with our account than classic self-monitoring accounts for the following two reasons. First of all, the PPF explains all of the data that earlier self-monitoring accounts explain, but goes much further. On the PPF, *all inputs* need to be ‘predicted’, not merely the self-produced ones. Second, as a few theorists have recently noted, ‘inputs’ include not merely external, sensory, inputs, but also interoceptive inputs (see Seth, 2013). So the brain also adopts ‘hypotheses’ to account for the presence of interoceptive information, including information that the subject might report as ‘anxiety’.
